# Novel Aspiration Thrombectomy and Blood Reinfusion System for Acute Intermediate-Risk Pulmonary Embolism: AVENTUS Trial Results

**DOI:** 10.1016/j.jscai.2025.103661

**Published:** 2025-05-02

**Authors:** Saher Sabri, Samuel Horr, Brian Stegman, Wissam A. Jaber, Michael A. Jolly, Christopher M. Huff, David O'Connor, Ahmad Younes, Nora E. Tabori, Peter P. Monteleone, Raghu Kolluri, Mehdi H. Shishehbor, Jun Li, AVENTUS Trial Investigators

**Affiliations:** a Divison of Interventional Radiology, MedStar Georgetown University Hospital, Washington, DC; b TriStar Centennial Medical Center, Nashville, Tennessee; c CentraCare Heart and Vascular Center, St. Cloud, Minnesota; d Division of Cardiology, Emory University School of Medicine, Atlanta, Georgia; e OhioHealth Research Institute, OhioHealth Riverside Methodist Hospital, Columbus, Ohio; f Hackensack University Medical Center, Hackensack, New Jersey; g ProMedica Toledo Hospital, Toledo, Ohio; h Ascension Texas Cardiovascular, UT Austin Dell School of Medicine, Austin, Texas; i Cardiovascular Service, OhioHealth Riverside Methodist Hospital & Syntropic Corelab, Columbus, Ohio; j University Hospitals Harrington Heart & Vascular Institute, Cleveland, Ohio

**Keywords:** aspiration thrombectomy, AVENTUS, blood return, functional outcomes, intermediate-risk pulmonary embolism, mechanical thrombectomy

## Abstract

**Background:**

Mechanical thrombectomy has become a first-line adjunctive therapy for anticoagulation in patients with acute intermediate-risk pulmonary embolism (PE). This prospective study evaluated the safety and efficacy of percutaneous mechanical aspiration thrombectomy with autologous blood reinfusion using the next-generation AVENTUS Thrombectomy System (Inquis Medical) in subjects with acute intermediate-risk PE.

**Methods:**

Subjects with acute intermediate-risk PE with symptoms ≤14 days were enrolled in this prospective, multicenter, single-arm study. Primary efficacy (defined as the change in the right ventricle to left ventricle [RV/LV] ratio from baseline) and safety defined as a composite rate of device-related major adverse events were both assessed at 48 hours postprocedure. Six-minute walk distance and quality of life were assessed at 30 days.

**Results:**

A total of 120 subjects were enrolled at 22 US sites. As compared to the baseline, the 48-hour RV/LV diameter ratio dropped significantly (0.47 ± 0.36; *P* < .0001), as did the refined modified Miller score (8.7 ± 5.5; *P* < .0001), reflecting a 35.9% reduction in clot burden. The mean estimated blood loss was 49.6 ± 38.9 mL, with no subjects requiring blood transfusion. Within 48 hours, no device-related major adverse events occurred. At 30 days, subjects exhibited a 132.9 m increase in 6-minute walk distance (*P* < .0001). PE-specific quality of life assessment at 30 days demonstrated decreased symptoms with a reduction of 25.3 points overall (*P* < .0001).

**Conclusions:**

This study confirms that thrombectomy and autologous blood reinfusion with the AVENTUS Thrombectomy System is a safe and effective primary treatment option in patients with acute intermediate-risk PE to improve RV function and reduce clot burden with minimal blood loss.

## Introduction

Acute pulmonary embolism (PE) results in 100,000 to 180,000 deaths per year,^1^ making it the third leading cause of cardiovascular mortality.^2^ Despite treatment with anticoagulation, the risk of death remains high.^3^^,^^4^ Furthermore, PE has been shown to impair the short-term and long-term quality of life (QoL) in survivors of post-PE syndrome or can result in chronic thromboembolic disease or pulmonary hypertension.^5^

Although catheter-directed thrombolysis has proven to be successful in reducing right ventricular (RV) dysfunction in intermediate and high-risk PE,^6^ it does not provide an immediate therapeutic result, and contraindications to thrombolytic therapy can limit its applicability.^7^ Recent evidence has supported the use of mechanical aspiration thrombectomy (MT) as an adjunctive therapy to anticoagulation in acute intermediate-risk PE to safely reduce clot burden and right ventricle to left ventricle (RV/LV) ratio.^8^, ^9^, ^10^ A recently published randomized controlled trial showed lower hemodynamic deterioration in patients undergoing MT versus catheter-directed thrombolysis but with no difference in mortality or major bleeding.^11^

Although MT has demonstrated effective clot burden reduction, it has not been without procedural complications, blood loss, and prolonged procedure time.^8^, ^9^, ^10^^,^^12^ Furthermore, functional and QoL outcomes in acute intermediate-risk PE following aspiration thrombectomy have not previously been prospectively reported in an investigational device exemption (IDE) trial.

The AVENTUS Thrombectomy System (ATS) (Inquis Medical) is a next-generation technology with additional features to reduce complications, blood loss, and procedural steps. The AVENTUS trial was designed to report clinical and procedural outcomes of this new technology in patients with acute intermediate-risk PE.

## Materials and methods

### Study design

The AVENTUS trial was a prospective, single-arm, multicenter IDE trial in which subjects with acute intermediate-risk PE underwent treatment and autologous filtered blood reinfusion with a percutaneous MT catheter and clot management system. The study was registered at ClinicalTrials.gov (NCT05907564) and was approved by a central institutional review board and local institutional review boards. All subjects who participated in the study signed written informed consent.

### Study population

Subjects aged 18 to 80 years with intermediate-risk acute PE and symptom duration ≤14 days and computed tomography pulmonary angiography (CTPA)-documented proximal PE were eligible for enrollment in the study. Intermediate-risk PE was defined as RV/LV ratio ≥0.9 per international guidelines.^7^^,^^13^

Subjects were required to be hemodynamically stable, with a systolic blood pressure ≥90 mm Hg without the need for vasopressors, and have a stable heart rate of <130 beats per minute prior to the procedure. Exclusion criteria included treatment with thrombolytics in the past 14 days, pulmonary hypertension with peak systolic pulmonary artery pressure (PAP) >70 mm Hg prethrombectomy, and actively progressing cancer treated with chemotherapeutics. The full list of inclusion and exclusion criteria is reported in Supplemental Table S1.

The targeted sample size was 120 subjects. In addition, each site was permitted to enroll and treat up to 2 roll-in subjects at its discretion. A subject was considered enrolled after informed consent was signed and once the aspiration catheter crossed the introducer sheath.

### Device description

**Figure 1:**
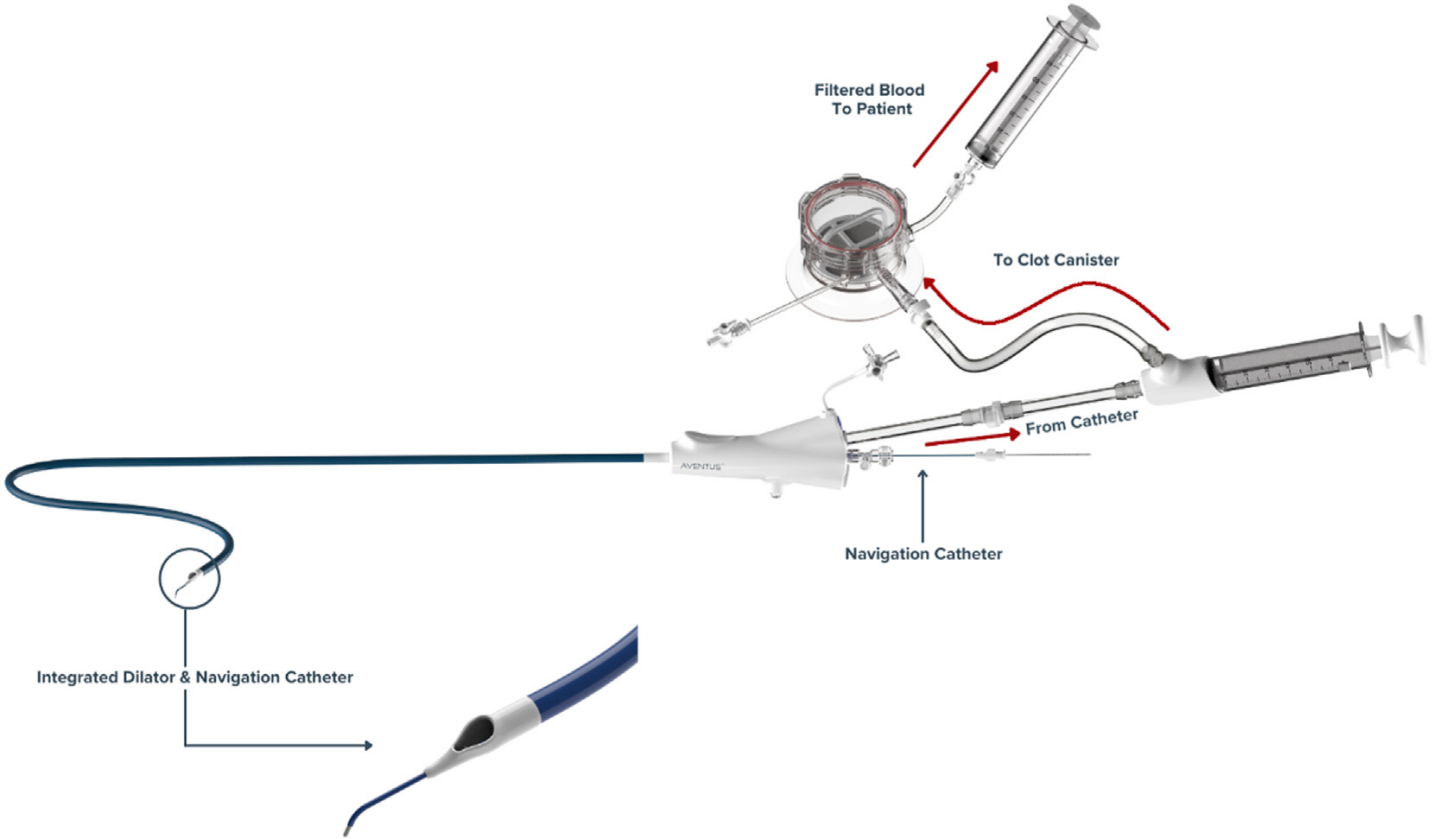
**AVENTUS Thrombectomy System.**

The ATS consists of an aspiration catheter, an aspiration syringe, and its associated blood filtration system (Figure 1). The AVENTUS 24F thrombectomy catheter is fully torqueable and features a radiopaque, atraumatic, angled tip without the need for a dilator to assist catheter advancement in tortuous anatomy and permit directional clot aspiration. In addition, the device includes an integrated navigation catheter, which assists with vessel selection and catheter navigation. Aspirated material is directed from the aspiration syringe into the in-line filtration system. A 1-way valve enables the direct transfer of aspirated contents to the clot management/blood filtration system without syringe disconnection, allowing rapid separation of thrombus from blood. Filtered blood is then returned via a transfusion filter at the access site, minimizing blood loss. The additional independent port allows real-time pressure monitoring and infusion of contrast without concern regarding clot embolization from the central lumen.

### Procedural details

All subjects underwent CTPA prior to the procedure to confirm study eligibility. Anticoagulation was administered per standard institutional practice in order to maintain an activated clotting time of >300 seconds. Femoral venous access was obtained and preceding PAP was measured. The 24F AVENTUS aspiration catheter was prepared and introduced into the femoral vein via a commercially available introducer sheath. Under fluoroscopic guidance, the catheter was advanced over a guide wire to the target location within the pulmonary vasculature. Once the catheter’s angled tip was positioned adjacent to the thrombus, manual aspiration was performed using the in-line aspiration syringe. Using the AVENTUS clot management system, the clot was separated and filtered blood was reintroduced into the patient’s venous access through a blood transfusion filter. After aspiration of thrombus from all target locations, the device and sheath were removed, and venous access site closure was performed per institutional practice. To better understand the efficacy and time efficiency of the device, the number of passes through the heart and catheter exchanges were documented.

### Efficacy end points

The primary efficacy end point was the change in RV/LV ratio from baseline (preprocedure) to 48 hours postprocedure as measured by CTPA. Additional efficacy end points included intensive care unit (ICU) and hospital length of stay, use of thrombolytics during and within 48 hours of the index procedure, and core laboratory-adjudicated change in refined modified Miller score from baseline to 48 hours postprocedure.

### Safety end points

The primary safety end point was the rate of device-related major adverse events (MAE) within 48 hours of the procedure. MAE were a composite of death, major bleeding, clinical deterioration, pulmonary vascular injury, or cardiac injury. See Supplemental Table S2 for definitions of the individual components of MAE. Additional safety end points included the rate of device-related serious adverse events and all-cause death, as well as symptomatic PE recurrence within 30 days of the procedure date.

### QoL end points

In addition to safety and efficacy, the AVENTUS trial followed the change in ambulatory distance within a 6-minute walk test from 48 hours postprocedure to 30 days, as well as the subject-reported change in QoL from baseline to 30 days postprocedure as measured by the pulmonary embolism-specific QoL (PEmb-QoL) questionnaire.^14^

### Independent oversight

An independent clinical events committee (CEC) adjudicated all potential MAE for the primary safety end point and a separate data safety and monitoring board provided safety oversight to the study. An independent medical monitor reviewed incoming safety data to ensure information was accurate and was responsible for CEC escalation decisions when applicable. An independent core lab reviewed all study CTPA for primary and additional efficacy end points.

### Statistical considerations

The primary efficacy end point of change in RV/LV ratio from baseline to 48 hours was compared to a minimum threshold performance goal of 0.20, consistent with or exceeding the efficacy performance goals of other published percutaneous thrombectomy devices.^8^^,^^10^ A paired *t* test with a 1-sided significance level of 2.5% was used for primary efficacy analysis.

The primary safety end point for the study of device-related MAE rate within 48 hours postprocedure was compared to a maximum threshold performance goal of 25%, which is also consistent with or more stringent than the safety performance goals of other published percutaneous thrombectomy devices.^8^, ^9^, ^10^ An exact binomial test with a 1-sided significance level of 2.5% was used for primary safety analysis.

The required sample size was determined using prior literature^8^^,^^10^ to estimate the expected performance of the ATS for both efficacy and safety aspects. Including a 10% assumption for attrition, it was determined that enrollment of 120 subjects was required to achieve an overall 80% power for the study.

The primary and observational safety end points were analyzed in the intention-to-treat (ITT) population, which consists of all subjects in whom the study device was introduced. The primary and observational efficacy end points were analyzed in the modified ITT (mITT) population, a subset of the ITT population which excluded subjects who received adjunctive treatment to reduce pulmonary artery (PA) clot burden up to 48 hours postprocedure. Efficacy-related end points that compared a mean change in values included mITT subjects with available paired data for both time points. Roll-in subjects are summarized separately and not included in outcomes analyses. However, for complete data transparency, primary safety and efficacy results on the roll-in subjects are provided.

The statistical analyses conducted for continuous variables included either means with standard deviation or medians with interquartile range. Categorical variables were summarized by frequencies and percentages. Unless explicitly stated, percentages used a denominator corresponding to the number of unique subjects contributing to the variable of interest. Exploratory analyses performed on additional end points were not adjusted for multiplicity.

All statistical analyses were performed using Stata statistical software version 17.0 or higher (StataCorp LLC).

## Results

A total of 120 ITT subjects with acute intermediate-risk PE were enrolled between September 2023 and January 2025 at 22 US sites. In addition, 7 of these sites enrolled a total of 10 roll-in subjects. Follow-up for primary safety was 100% (120/120), and 97.5% (116/119) of mITT subjects had follow-up for primary efficacy at 48 hours.

Baseline subject demographics and clinical characteristics are listed in Table 1 for the ITT cohort, as well as the ITT and roll-in combined cohort. The majority were men (55.8%); the average age was 58.8 years. Key medical history included hypertension (54.2%), concomitant deep vein thrombosis (84.3%, 86/102), prior PE (8.3%), and history of cancer (9.2%). Based on the modified Bova score^15^ for PE complications, 60.0% of subjects were within the intermediate-risk (stage II) category, whereas 27.5% were classified as high-risk (stage III). A total of 87.4% (104/119) of subjects had elevated troponin or brain natriuretic peptides. In addition, central clot distribution was present in 93.3% of subjects, and bilateral clot in 99.2% of subjects.

**Table 1:** Patient demographics and baseline clinical characteristics.

Characteristic	ITT^a^ (n = 120)	ITT and roll-in^a^ (n = 130)
Age, y	58.8 ± 12.3 (120)	59.2 ± 12.3
Male sex	55.8 (67/120)	57.7% (75/130)
Race		
White	80.2 (93/116)	79.4% (100/126)
Black or African American	17.2% (20/116)	18.3% (23/126)
Other	2.6% (3/116)	2.4% (3/126)
Ethnicity		
Not Hispanic or Latino	90.8% (108/119)	90.7% (117/129)
Hispanic or Latino	5.9% (7/119)	6.2% (8/129)
Not reported	3.4% (4/119)	3.1% (4/129)
Body mass index, kg/m^2^	35.6 ± 7.4 (115)	35.9 ± 8.5 (125)
Prior pulmonary embolism	8.3% (10/120)	8.5% (11/130)
History of CAD	4.2% (5/120)	5.4% (7/130)
Prior DVT	10.0% (12/120)	10.0% (13/130)
Concomitant DVT	84.3% (86/102)	83.6% (92/110)
Hypertension	54.2% (65/120)	55.4% (72/130)
Any cancer	9.2% (11/120)	10.8% (14/130)
Active cancer^b^	3.3% (4/120)	3.1% (4/130)
Elevated troponins or BNP^c^	87.4% (104/119)	86.8% (112/129)
Modified Bova score		
I	12.5% (15/120)	12.3% (16/130)
II	60.0% (72/120)	62.3% (81/130)
III	27.5% (33/120)	25.4% (33/130)
Clot location^d^		
Central	93.3% (111/119)	93.8% (120/128)
Bilateral	99.2% (119/120)	98.4% (127/129)

Values are % (n/N) or mean ± SD.

BNP, brain natriuretic peptides; CAD, coronary artery disease; DVT, deep vein thrombosis.

a Lower denominators reflect the number of subjects with available data.

b Per exclusion criteria, patients with active cancer were eligible for enrollment if the cancer was not known to be actively progressing and treated with chemotherapeutics.

c Values of 90 pg/mL (BNP), 500 pg/mL (N-terminal pro-BNP), 0.03 ng/mL (troponin I), 0.1 ng/mL (troponin T).

d Central clot was defined as a clot in the main pulmonary artery (PA) trunk or in the main right PA or in the main left PA.

### Procedural characteristics and outcomes

Procedural and postoperative details are reported in Table 2 for the ITT cohort and the ITT and roll-in combined cohort. Access was obtained via the right femoral vein in 94.2% and the left femoral vein in 5.8% of subjects. The majority of procedures were performed under conscious sedation 89.2% (107/120) with a median catheter dwell time of 39.5 minutes. Catheter dwell time was defined as the total procedural time during which the aspiration catheter was introduced into and withdrawn from the subject inclusive of tracking, aspirations, and autologous blood reinfusion. There were no device malfunctions and no use of adjunctive thrombolytics during the procedure, or at any time during the 30-day follow-up. In 60.8% (73/120) of subjects, the procedure was completed with a single pass through the heart. On average, mean PAP decreased by 4.8 mm Hg (*P* < .0001), from 28.5 mm Hg preaspiration to 23.7 mm Hg postaspiration. This decrease was more pronounced in the 76 subjects who presented with a mean PAP >25 mm Hg, with a reduction in mean PAP of 6.1 mm Hg in this subgroup. One subject had adjunctive thrombectomy treatment during the index procedure. The mean hospital length of stay was 2.6 days. The mean ICU stay was 0.8 days, and 31.1% of subjects had an ICU stay of 1 day or greater.

**Table 2:** Procedure characteristics.

Characteristic	ITT (n = 120)	ITT and roll-in (n = 130)
Access site		
Right femoral vein	94.2% (113/120)	93.8% (122/130)
Left femoral vein	5.8% (7/120)	6.2% (8/130)
Sedation during procedure		
Conscious sedation	89.2% (107/120)	90.0% (117/130)
General anesthesia	5.0% (6/120)	4.6% (6/130)
Local anesthesia	5.8% (7/120)	0.0% (0/130)
Had autologous blood reinfusion	99.2% (119/120)	99.2% (129/130)
Thrombectomy and autologous blood reinfusion time, min	39.5 (31.0-59.5)	40.5 (31.0-61.0)
Blood loss (net), mL	49.6 ± 38.9 (119)	50.2 ± 40.0 (129)
Mean PAP preaspiration, mm Hg^a^	28.5 ± 7.8 (118)	28.7± 7.9 (128)
Mean PAP postaspiration, mm Hg	23.7 ± 7.8 (119)	23.8 ± 7.7 (129)
Change in mean PAP, mm Hg	−4.8 ± 5.5 (117)^c^	−4.8 ± 5.5 (127)
Change in mean PAP in pulmonary HTN subjects, mm Hg^b^	6.1 ± 6.0 (75)	6.2 ± 6.0 (82)
Thrombolytic use within 48 h postprocedure	0% (0/120)	0% (0/130)
Adjunctive thrombectomy treatment within 48 h postprocedure	0.8% (1/120)	1.5% (2/130)
Required blood transfusion	0% (0/120)	0% (0/130)
Length of hospital stay, d	2.6 ± 2.2 (119)	2.6 ± 2.2 (128)
Percent with ICU stay ≥1 d	31.1% (37/119)	29.7% (38/128)
Length of ICU stay, d	0.8 ± 1.8 (119)	0.8 ± 1.8 (128)

Values are % (n/N), median (IQR), or mean ± SD (n).

HTN, hypertension; ICU, intensive care unit; ITT, intention-to-treat; PAP, pulmonary artery pressure.

a Sample sizes shown for preaspiration and postaspiration mean PAP includes all subjects with data. The sample size shown for changes includes only subjects with data for both time points (ie, paired analyses).

b Pulmonary HTN is defined as mean PAP >25 mm Hg by right heart catheterization.

c Comparison of post-aspiration to pre-aspiration mean PAP, *P* < .0001.

All but one subject had autologous blood filtered and reinfused during the procedure via the AVENTUS clot management system, resulting in a net estimated blood loss (EBL) of 49.6 ± 38.9 mL. No subjects required a blood transfusion during the procedure or through 48 hours postprocedure.

### Efficacy end points

The mITT analysis population (n = 116) included the subset of ITT (N = 120) subjects with paired baseline and 48-hour core lab adjudicated RV/LV ratio and in whom the thrombectomy system was the only treatment to reduce clot burden through 48 hours. In these subjects, the RV/LV ratio at 48 hours postprocedure decreased from 1.57 ± 0.40 at baseline to 1.10 ± 0.20 at 48 hours following thrombectomy and blood reinfusion, a 0.47 ± 0.36 (*P* < .0001) decrease (Central Illustration). The 0.47 mean change in RV/LV ratio exceeded the performance goal of 0.20 (*P* < .0001) and thus met the primary efficacy end point. Although not included in the primary efficacy end point calculation, the results for the roll-in subjects treated without the use of adjunctive treatment to reduce clot burden (n = 9) were consistent with a mean RV/LV ratio decrease of 0.49 ± 0.36 from baseline (1.53 ± 0.44) to 48 hours (1.04 ± 0.18).

**Central Illustration:**
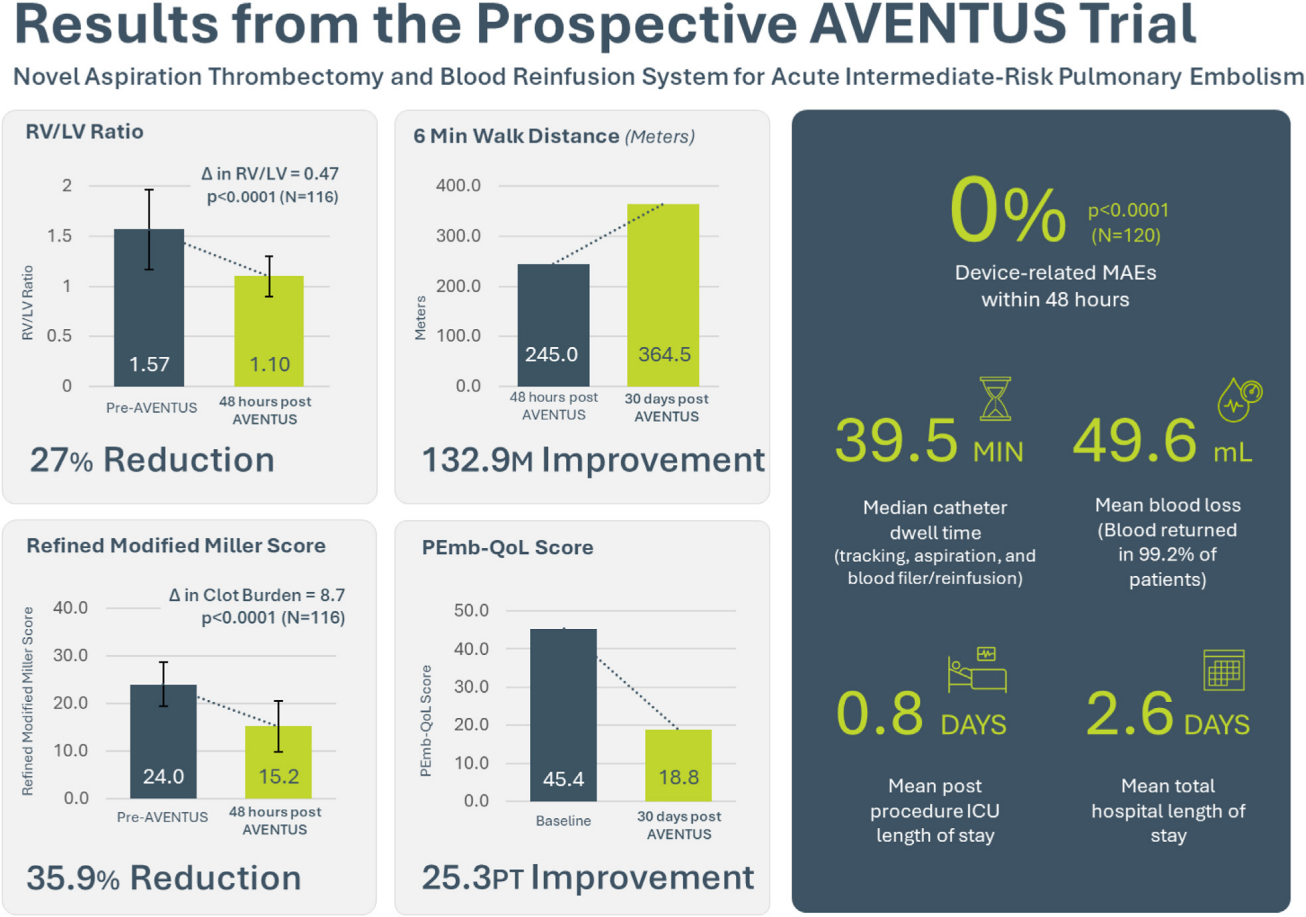
**Key results from the prospective AVENTUS trial.** ICU, intensive care unit; MAE, major adverse event; RV/LV, right ventricle to left ventricle; PEmb-QoL, pulmonary embolism-specific quality of life.

**Figure 2:**
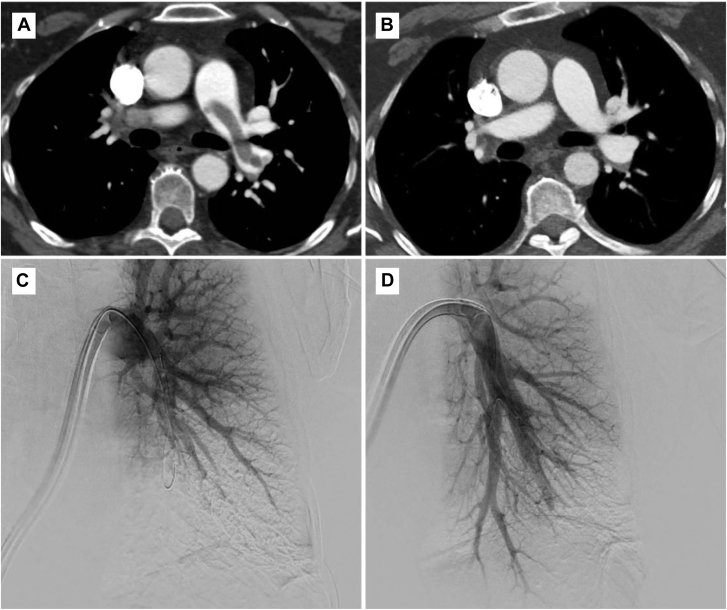
**Computed tomography (CT) scans and angiograms illustrating a reduction in clot burden. (A)** Preprocedure CT, (**B**) 48 hours postprocedure CT, (**C**) left preaspiration angiogram, and (**D**) left postaspiration angiogram. Case images provided by Dr Samuel Horr (TriStar Centennial Medical Center).

The mean refined modified Miller score decreased significantly by 8.7 points (*P* < .0001) at 48 hours (15.2) from baseline (24.0), representing a 35.9% reduction in clot burden. Figure 2 is an example of extracted PE from 1 of the study subjects.

### Safety end points

No subjects in the ITT population (0.0%, 0/120) experienced a device-related MAE within 48 hours of the procedure, meeting the prespecified safety performance goal of less than 25% device-related MAE rate (*P* < .0001). Similarly, there were no device-related MAE in the roll-in population (0.0%, 0/10).

There were no device-related MAE or serious adverse events within 30 days as adjudicated by an independent CEC, as shown in Table 3, which includes ITT as well as the combined ITT and roll-in. There were 3 subjects with nondevice-related MAE occurring within 48 hours, all during the index procedure. Two of the 3 subjects experienced clinical deterioration. The first subject with preexisting chronic pulmonary hypertension became hypoxic and hypotensive post thrombectomy, developed cardiac arrest with pulseless electrical activity, and required emergency intubation and veno-arterial extracorporeal membrane oxygenation support. This subject continued to decline until death (adjudicated by CEC as nondevice-related) at 16 days postprocedure. The second subject experienced tachypnea and hypoxic arrest with pulseless electrical activity and required intubation prior to PA catheterization and introduction of the thrombectomy device. Successful thrombectomy was performed with rapid improvement in hemodynamics. The subject continued to improve, was extubated on postprocedure day 1, and discharged in stable condition 7 days later. A third subject experienced a nondevice-related hemoptysis event after a preexisting cough progressed during early aspirations and became productive of blood, requiring emergent intubation. A bronchoscopy and repeat angiography did not show evidence of active bleeding or extravasation. No major interventions were required, and the subject was extubated 2 days postprocedure and discharged 3 days later.

**Table 3:** Safety and efficacy end points.

Outcome^a^		
Primary efficacy	mITT	mITT and roll-in
Baseline RV/LV ratio	1.57 ± 0.40 (119)	1.57 ± 0.40 (127)
48-h RV/LV ratio	1.10 ± 0.20 (116)	1.10 ± 0.20 (125)
Change in RV/LV ratio^b^	−0.47 ± 0.36 (116)	−0.47 ± 0.36 (125)
Percent reduction in RV/LV ratio	27.0% ± 17.1% (116)	27.1% ± 17.0% (125)
Reduced clot burden	mITT	mITT and roll-in
Baseline refined modified Miller score	24.0 ± 4.6 (118)	24.1 ± 4.7 (126)
48-h refined modified Miller score	15.2 ± 5.4 (116)	15.3 ± 5.5 (124)
Change in refined modified Miller score^c^	−8.7 ± 5.5 (116)	−8.8 ± 5.6 (124)
Percent reduction in refined modified Miller score	35.9% ± 21.6% (116)	35.9% ± 21.7% (124)
Primary safety	ITT	ITT and roll-in
Device-related MAE within 48 h^d^	0% (0/120)	0% (0/130)
Any MAE within 48 h	2.5% (3/120)	2.3% (3/130)
Nondevice-related major bleed	0.8% (1/120)	0.7% (1/130)
Nondevice-related clinical deterioration	1.7% (2/120)	2.3% (3/130)
30-d device-related MAE or SAE	0% (0/113)	0% (0/123)
PE recurrence	ITT	ITT and roll-in
Symptomatic PE recurrence within 30 d	0.9% (1/113)	0.8% (1/123)

Values are % (n/N) or mean ± SD (n).

ITT, intention-to-treat; MAE, major adverse event; mITT, modified intention-to-treat; PE, pulmonary embolism; RV/LV, right ventricle to left ventricle; SAE, serious adverse event.

a Sample sizes shown for baseline 48-hour RV/LV and refined modified Miller score include all subjects with data. Sample sizes shown for changes include only subjects with data for both time points (ie, paired analyses).

b *P* value for change in RV/LV ratio from baseline to 48 hours vs the prespecified performance goal, *P* < .0001.

c Comparison of 48 hour to baseline refined modified miller score, *P* < .0001.

d *P* value for the percentage of ITT subjects experiencing a device-related MAE within 48 hours vs the prespecified performance goal, *P* < .0001.

There was 1 additional subject who had nondevice-related MAE through 30 days. This subject experienced a major bleed starting on day 10 due to significant vaginal bleeding and arterial access–related bleed (for diagnostic uterine artery angiogram) related to recently diagnosed cervical cancer. There was 1 incident (0.9%) of recurrent PE within 30 days.

### QoL end points

In a paired analysis (Table 4), the mean 6-minute walk distance (6MWD) increased significantly (*P* < .0001) by 132.9 m at 30 days compared to 48 hours postprocedure, with the proportion of subjects walking over 350 m rising from 17.3% at baseline to 55.8% at 30 days. Subjects who had both 48-hour and 30-day data for 6MWD were included in the analysis. Several subjects did not have 6MWD data at both time points due to clinical limitations (eg, nonambulatory) or logistics associated with research site operations (eg, weekend staffing shortages). Subject-reported QoL, measured by the PEmb-QoL questionnaire, improved from 45.4 at baseline to 18.8 at 30 days and showed a significant decrease in score of 25.3 points (*P* < .0001).

**Table 4:** QoL outcomes.

Outcome	mITT	mITT and roll-in
48-h 6MWD in meters (N)^a^	245.0 ± 136.0 (81)	246.8 ± 135.3 (88)
Subjects walking >350 m (n/N)	17.3% (14/81)	17.0% (15/88)
30-d 6MWD in meters (N)^a^	364.5 ± 162.5 (95)	362.9 ± 157.6 (103)
Subjects walking >350 m (n/N)	55.8% (53/95)	54.4% (56/103)
30-d change in distance from 48 h (m)	132.9 ± 198.8 (74)^b^	129.6 ± 195.8 (80)
Baseline PEmb-QoL score^a^	45.4 ± 22.9 (117)	45.3 ± 22.7 (126)
30-d PEmb-QoL score^a^	18.8 ± 20.6 (106)	18.4 ± 20.3 (114)
Change from baseline	−25.3 ± 25.7 (104)^c^	−25.9 ± 25.4 (112)

Values are % (n/N) or mean ± SD (n).

6MWD, 6-minute walk distance; mITT, modified intention-to-treat; PEmb-QoL, pulmonary embolism-specific quality of life; QoL, quality of life.

a Sample size shown for 48 hour/30-day 6MWD and baseline/30-day QoL includes all subjects with data. The sample size shown for changes includes only subjects with data for both time points (ie, paired analyses).

b Comparison of 30 day to 48 hour distance walked, *P* < .0001.

c Comparison of 30 days to baseline PEmb-QoL score, *P* < .0001.

## Discussion

The AVENTUS trial reports the safety and efficacy of the ATS, with the incorporation of autologous blood reinfusion, for the treatment of patients with acute intermediate-risk PE. The 0.47 decrease (*P* < .0001) in RV/LV ratio at 48 hours exceeded the 0.20 performance goal for this study, meeting the study’s primary end point and demonstrating efficacy consistent with reported results of other percutaneous commercially available thrombectomy devices.^8^, ^9^, ^10^

The AVENTUS trial met the primary safety end point, with no (0%) device-related MAE at 48 hours compared to the performance goal of 25% (*P* < .0001). Three subjects had 48-hour MAE reported, adjudicated by the CEC as not device-related, including 1 nonfatal bleeding event. It is notable that the reported safety and efficacy results were achieved despite no prior in-human experience with the device. Up to 2 roll-in subjects per investigational site were permitted (at the investigator’s discretion), but only 7 out of 22 sites enrolled in the roll-in cohort for a total of 10 subjects. In the majority of cases, once the catheter was delivered into the PA, no additional exchanges were required. This suggests significant maneuverability related to the novel catheter design, resulting in selective catheterization and delivery without the need for multiple exchanges or adjunctive dilator use. The median catheter dwell time including blood filtration and reinfusion was 39.5 minutes, which compares favorably to the recently published PEERLESS study where the mean catheter dwell time was 47.9 minutes.^11^ This device also showed a significant clot reduction of 35.9% at 48 hours using each subject as their own control. Recognizing the differences in scoring systems and methods of calculation, the outcomes of this trial rank among the highest percent reduction in the literature for contemporary devices, which ranged from 9.3% to 35.9%.^8^, ^9^, ^10^^,^^15^, ^16^, ^17^

This trial is the first prospective IDE study to assess the safety of autologous blood reinfusion alongside MT for this patient population. As the study device incorporated blood filtration and reinfusion features, it was anticipated that blood loss would be low, as verified by less than 50 mL of mean EBL per patient. A distinct advantage of an in-line filtration system is that it minimizes the loss of blood during filter cleaning. As such, the EBL associated with the ATS is lower than other large-bore thrombectomy systems that require separate filter-and-cleaning procedures to reinfuse blood, as was demonstrated in the mean EBL of 88 mL in the PEERLESS study.^18^ Furthermore, MT with a sustained vacuum pump does not permit blood reinfusion, which resulted in greater blood loss with 27% of patients in the EXTRACT-PE study having EBL >400 mL.^10^ In the AVENTUS trial, controlled syringe-based aspiration was used, which may minimize the mechanical stress on blood. The quality of the reinfused blood was verified by the absence of reinfusion-associated adverse events and no blood transfusion requirements.

Over the last 2 decades, several reports have revealed that up to 50% of long-term PE survivors suffer from dyspnea, reduced health-related QoL, and/or impaired exercise capacity,^19^, ^20^, ^21^, ^22^ a constellation of symptoms that has been coined “post-PE syndrome.”^5^ Although this syndrome has been recently characterized with long-term (≥12 months) exercise functional measures (eg, by 6MWD or cardiopulmonary exercise test)^21^, ^22^, ^23^ following PE treated by anticoagulation therapy,^21^, ^22^, ^23^ there is little published on shorter-term exercise outcomes following PE. Importantly, even short-term functional outcomes such as 6MWD at 1 month are predictive of pulmonary function in the longer-term, with distances walked generally increasing over time.^22^ Similarly, the PE-specific and validated QoL questionnaire, PEmb-QoL,^14^^,^^24^ has been studied in mainly longer-term (>12 months) follow-up in patients with PE treated with anticoagulation therapy.^22^^,^^25^ Of note, the recently published FLASH registry did show an improvement in 6MWD and median PE QoL score at 30 days and 6 months.^26^

Although this study is limited to short-term 30-day follow-up, we demonstrated significant increases in exercise capacity over the study period, as measured by 6MWD at 1 month compared to 48 hours postthrombectomy (132.9 m, *P* < .0001), as well as improvement in QoL, as demonstrated by the reduction of symptom score in the PEmb-QoL assessment (25.6, *P* < .0001) as compared to baseline. The impact of catheter-directed therapies on QoL and functional outcomes compared to anticoagulation alone needs to be evaluated. Randomized, controlled trials including PE-TRACT (NCT05591118), HI-PEITHO (NCT04790370), STORM-PE (NCT05684796), and PEERLESS II (NCT06055920) are underway to evaluate the role of catheter-based therapies against anticoagulation in longer-term patient QoL and functional status.

As the study lacked randomization or a comparator arm, comparisons with anticoagulation therapy alone or alternative mechanical thrombectomy devices are limited. Additionally, the study population included patients with acute intermediate-risk PE; therefore, results cannot be directly applied to high-risk/massive PE. Finally, this first-in-human IDE trial evaluated short-term follow-up at 30 days, which limits the conclusions that can be made about longer-term clinical, functional, and QoL outcomes.

## Conclusion

This study confirms the safety and efficacy of the ATS for percutaneous aspiration thrombectomy and autologous blood reinfusion in patients diagnosed with acute intermediate-risk PE. A significant improvement in RV/LV ratio and clot burden reduction was demonstrated, accompanied by minimal procedural blood loss.
