# Supplementary material for: Novel Aspiration Thrombectomy and Blood Reinfusion System for Acute Intermediate-Risk Pulmonary Embolism: AVENTUS Trial Results
Source: J Soc Cardiovasc Angiogr Interv. 2025 May 2;4(7):103661. doi: 10.1016/j.jscai.2025.103661 (PMC12418419; doi:10.1016/j.jscai.2025.103661)
Supplement: Supplementary Table 1 [file mmc1.docx]

**Supplemental Table S1 – AVENTUS Trial Full Inclusions/Exclusion Criteria**

**Inclusion Criteria:**

1. Age ≥ 18 and ≤ 80 years ​
2. Subject or legally authorized representative (LAR) was willing and able to provide written informed consent prior to receiving any non-standard of care protocol specific procedures
3. Subject was willing and able to comply with all protocol required follow-up visits
4. PE symptom(s) duration ≤ 14 days​ from index procedure
5. PE diagnosis ≤ 48 hours prior to index procedure
6. CTA evidence of proximal PE (filling defect in at least one main or lobar pulmonary artery based on Investigator determination)​
7. CTA evidence of dilated RV with an RV/LV ratio of ≥ 0.9 at baseline based on Investigator determination
8. Systolic blood pressure ≥ 90mmHg without need for vasopressors (initial SBP ≥ 80 mmHg was allowed if the pressure recovered to ≥ 90 mmHg with intravenous fluids prior to the index procedure)
9. Stable heart rate <130 BPM prior to the index procedure
10. Subject was deemed medically eligible for interventional procedure(s), per institutional guidelines and clinical judgment

**Exclusion Criteria:**

1. Prior PE ≤ 180 days from index procedure
2. Concurrent hospitalization for other condition(s)
3. Thrombolytic use ≤ 14 days of baseline CTA​
4. Pulmonary hypertension with peak pulmonary artery systolic pressure > 70 mmHg by right heart catheterization​​
5. FiO2 requirement > 40% or > 6 LPM to keep oxygen saturation > 90%​
6. Hematocrit < 28% within 6 hours of index procedure
7. Platelets < 100,000/μL​
8. Serum creatinine > 1.8 mg/dL​
9. International normalized ratio (INR) > 3​
10. Presence of intracardiac lead in the right ventricle or right atrium placed < 180 days prior to the index procedure
11. Cardiovascular or pulmonary surgery ≤ 7 days​ prior to the index procedure
12. Actively progressing cancer​ treated by chemotherapeutics
13. Known bleeding diathesis or coagulation disorder​
14. Left bundle branch block​
15. History of severe or chronic pulmonary arterial hypertension​
16. History of chronic left heart disease with left ventricular ejection fraction ≤ 30%​
17. History of uncompensated heart failure​
18. History of underlying lung disease that is oxygen dependent​
19. History of chest irradiation​
20. History of heparin-induced thrombocytopenia (HIT)​
21. Any contraindication to systemic or therapeutic doses of heparin or anticoagulants​
22. Known anaphylactic reaction to radiographic contrast agents that could not be pretreated​
23. Imaging evidence or other evidence that suggested, in the opinion of the Investigator, the subject was not appropriate for aspiration thrombectomy intervention such as the inability to navigate to target location, predominantly chronic clot or non-clot embolus
24. Life expectancy of < 90 days, as determined by Investigator​
25. Female who was pregnant or nursing​
26. Concurrent participation in another investigational drug or device treatment study​ that had not reached the primary endpoint or the Investigator felt would impact their ability to participate in this clinical trial
27. Subject had known residual Iliac deep vein thrombosis (DVT), inferior vena cava (IVC) clot or clot in transit (right atrium and/or right ventricular)
28. Subject on extracorporeal membrane oxygenation (ECMO)
