# Supplementary material for: Novel Aspiration Thrombectomy and Blood Reinfusion System for Acute Intermediate-Risk Pulmonary Embolism: AVENTUS Trial Results
Source: J Soc Cardiovasc Angiogr Interv. 2025 May 2;4(7):103661. doi: 10.1016/j.jscai.2025.103661 (PMC12418419; doi:10.1016/j.jscai.2025.103661)
Supplement: Supplementary Table 2 [file mmc2.docx]

**Supplemental Table S2 – Study Definitions of Major Adverse Events**

| Term | Definition |
| --- | --- |
| Bleeding | Defined as loss of blood from the vascular system. Bleeding was classified by the GUSTO (Global Utilization of Streptokinase and Tissue Plasminogen Activator for Occluded Coronary Arteries) bleeding criteria:  Major bleeding includes GUSTO Severe/life threatening and Moderate categories:  GUSTO Severe:   - Intracranial Hemorrhage - Fatal/life threatening bleeding - Resulting in substantial hemodynamic compromise requiring intervention   GUSTO Moderate:   - Requiring blood transfusion but did not result in hemodynamic compromise   Minor bleeding includes GUSTO Mild bleeding:   - GUSTO Mild bleeding that does not meet above criteria |
| Clinical Deterioration | Also referred to hemodynamic collapse, is defined as having one of the following:   - need for cardiopulmonary resuscitation, intubation, vasopressors, ECMO/ECLS; or systolic blood pressure (SBP) <90 mmHg for at least 15 minutes, or   drop of SBP by at least 40 mmHg for at least 15 minutes with signs of end-organ hypoperfusion (cold extremities or low urinary output <30 mL/h or altered mental status); or   - need for catecholamine administration to maintain adequate organ perfusion and a SBP >90 mmHg (including dopamine at the rate of >5 micrograms/kg per minute). |
| Cardiac Injury | The following events are examples of possible cardiac injury:  • Acute heart failure  • Acute myocardial infarction  • Arrhythmia requiring intervention  • Cardiac hematoma  • Tricuspid or Pulmonic valve damage |
| Device-related Death | Device related death is defined as any death directly related to the device not performing as expected. Device related death would include death from:  • Vascular or cardiovascular injury  • Device malfunction  • Device-induced cardiac arrhythmia  • Worsening pulmonary or right heart function (exclusive of worsening from recurrent PE) |
| Pulmonary Vascular Injury | Pulmonary vascular injury may include the following events occurring in the pulmonary vasculature:  • Arterial Venous Fistula  • Dissection  • Hemorrhage  • Intimal flap  • Perforation  • Rupture  • Thromboembolic occlusion resulting in permanent damage i.e., infarction |
